# Competitive Fitness of Cytomegalovirus Mutants Bearing Changes in the UL56 Terminase Subunit, Associated with Letermovir-Resistance, in Presence and Absence of Antivirals

**DOI:** 10.3390/v18070779

**Published:** 2026-07-15

**Authors:** Graciela Andrei, Sarah Gillemot, Robert Snoeck

**Affiliations:** Rega Institute for Medical Research, Department of Microbiology, Immunology and Transplantation, KU Leuven, 3000 Leuven, Belgium

**Keywords:** cytomegalovirus, letermovir, terminase, DNA polymerase, drug resistance, competitive fitness, antiviral drugs, dual-infection competition assays, next-generation sequencing, antiviral drug susceptibility

## Abstract

Letermovir (LMV), a cytomegalovirus (CMV) terminase inhibitor, has a potentially low genetic barrier to the emergence of resistance, with single mutations in the terminase subunits (UL51, UL56, UL89) being associated with LMV resistance (LMV-R). We determined the fitness of LMV-R viruses (UL56 C325F/W/Y or M236V mutants) with different DNA polymerase (pol) (UL54) mutants in the presence and absence of anti-CMV drugs in dual-infection competition assays. After 7 days of growth, viral variants were quantified by targeted sequencing of the UL54 or UL56 genes by next-generation sequencing. Without antivirals, the UL56 C325F mutant was equally fitted as the wild-type virus, in contrast to C325W/Y mutants that had a reduced replication capacity. The UL56 C325F mutant showed the highest replication capacity among all terminase mutants tested when grown in competition with wild-type virus under LMV. The UL56 terminase mutants gained replication capacity when grown in competition with DNA pol mutant viruses under LMV pressure, with the terminase mutant UL56 C325F being able to overgrow various DNA pol mutants. Furthermore, the UL56 C325F mutant showed the highest replication capacity when grown in competition with another UL56 terminase mutant. Our results are in line with the C325F UL56 mutant being the most frequent LMV-R mutation identified in the clinic.

## 1. Introduction

Letermovir (LMV, Prevymis^®^) was approved in 2018 for prophylaxis of cytomegalovirus (CMV) infection and disease in adult allogeneic hematopoietic stem cell transplant (HSCT) CMV-seropositive recipients (R+) [1]. Since its introduction, letermovir became the standard for preventing clinically significant CMV infection in this patient population [2]. Currently, the use of valganciclovir (VGCV), ganciclovir (GCV), or intravenous immuno-globulin as prophylaxis against CMV reactivation is generally not recommended. Letermovir prophylaxis should be started no later than day 28 after transplantation and should be maintained for at least 100 days post-HSCT. Extended prophylaxis for at least 200 days after transplantation should be considered among patients presenting high risk for CMV disease [3]. Letermovir secondary prophylaxis can be envisaged after successful treatment of a CMV reactivation in patients at high-risk for CMV disease or in patients who did not receive CMV primary prophylaxis and were successfully treated for reactivated CMV disease [2,4,5].

In June 2023, the Food and Drug Administration (FDA) approved LMV for the prevention of CMV disease in high-risk adult kidney transplant recipients, CMV-seropositive donors/CMV-seronegative recipients (D+/R−) [6]. This expansion of LMV use marked the official approval for solid organ transplant (SOT) prophylaxis. LMV is effective as primary or secondary prophylaxis in SOT patients [7,8,9]. For kidney, liver, pancreas, or heart transplant recipients who are D+/R− where prophylaxis is used, switching to LMV prophylaxis in case of intolerance to VGCV or GCV can be considered [10]. The use of LMV monotherapy for the treatment of refractory/resistant (R/R) CMV infection is not recommended because of the low barrier to the development of resistance of the drug in a CMV infection setting [11,12,13].

Emergence of LMV resistance (LMV-R) during primary prophylaxis in HSCT recipients has been reported at a low incidence (below 2% in phase 3 clinical trials) [14], though it appeared to be higher when used for secondary prophylaxis [5]. Specific risk factors for the emergence of LMV-R during primary or secondary prophylaxis include temporary suspension of treatment, malabsorption, or any other cause leading to low drug concentrations [14,15,16]. In case reports and uncontrolled series, development of LMV-R correlated with increased morbidity and mortality [12,17,18].

Letermovir uniquely targets the UL56 subunit of the CMV-terminase complex, formed by three subunits, i.e., UL51, UL56, and UL89 [11,19,20]. Therefore, LMV does not show cross-resistance with CMV DNA polymerase (pol) inhibitors [i.e., GCV, cidofovir (CDV, HPMPC)], and foscarnet (foscavir, PFA) [20,21,22]. Resistance to DNA pol inhibitors emerges due to mutations in the UL97 protein kinase (PK) (involved in the activation of GCV) and/or in the viral DNA pol (the drug target of GCV, CDV, and PFA) [11]. Resistance to LMV is associated with changes in one of the three terminase subunits, and mutations at amino acid positions 236, 257, 325, and 329 in the UL56 gene reduce LMV efficacy by >3000-fold. Substitutions at codon 325 (C325Y/F/W/R) are the most frequent, in particular C325F [23,24].

Mutations that confer LMV-R have been demonstrated to have minimal to low impact on growth fitness compared to wild-type virus in mono-infection experiments [24]. However, the gold standard for estimating the relative replication capacity of a viral strain is a dual-infection competition assay, in which drug-resistance (drug-R) and reference strains are grown together to ensure equivalent growth conditions, and viral variants are quantified by qPCR (quantitative polymerase chain reaction), by the sequencing of individual viral clones, and more recently by deep-sequencing [25,26,27,28,29]. To date, no such studies have been performed in CMV; the fitness of CMV drug-R mutants has compared viral growth in mono-infections [24,30,31]. However, this approach fails to mimic the situation in the clinic where a genetic CMV diversity is found and heterogeneous populations of drug-R virus exits [13,32,33,34,35,36].

In some competitive fitness studies, performed mostly with RNA viruses, bulk or clonal sequencing determined the ratio of wild-type and mutant viruses. In other studies, a small reporter gene was introduced into the reference viral genome as a tag to facilitate the differential quantification of the two competing viruses [36]. In our laboratory, we have developed a new methodology for evaluating competitive fitness of drug-R herpesviruses that is based on the use of next-generation sequencing (NGS) for the analysis of viral populations, as this technology can detect changes in the relative ratio of the distinct viruses [37,38,39,40].

In this study we evaluated the replication capacity of LMV-R viruses in competition with wild-type virus, and with well-characterized DNA pol mutants in the presence and absence of drug pressure. Also, competition between two different LMV-R viruses was investigated. Our studies showed the effects of antiviral therapy and competition on viral fitness and on the dynamics of the viral populations.

## 2. Materials and Methods

### 2.1. Cells

Human embryonic lung (HEL) 299 cells (CCL-137 ™) were purchased from ATCC (Manassas, Virginia). (HEL) fibroblasts are human diploid lung cells derived from normal fetal lung tissue, extensively employed in virology. HEL cells were maintained in Dulbecco’s modified Eagle’s medium (DMEM, Thermo Fisher Scientific, Merelbeke, Belgium) supplemented with 8% fetal bovine serum (FBS).

### 2.2. Compounds

The sources of compounds were as follows: adefovir (ADV, PMEA, 9-(2-phosphonylmethoxyethyl)adenine) and cidofovir (CDV, HPMPC, (*S*)-1-(3-hydroxy-2-phosphonylmethoxypropyl)cytosine) [Gilead sciences, Foster City, CA, USA]; acyclovir (ACV) and foscarnet (phosphonoformic acid, foscavir, PFA) [Merck, Hoeilaart, Belgium]; ganciclovir (GCV, cymevene) [Roche, Basel, Switzerland]. PMEDAP [9-(2-phosphonylmethoxyethyl)-2,6-diaminopurine] and HPMPA [(*S*)-9-(3-Hydroxy-2-phosphonomethoxypropyl)adenine] were kindly provided by Dr Marcela Krečmerová, Institute of Organic Chemistry and Biochemistry, Czech Academy of Sciences, Prague, Czech Republic. Letermovir (LMV) was purchased from Cayman Chemical, Ann Arbor, MI, USA.

### 2.3. Viruses

The UL56 C235F, C325Y, C325W, and V236M mutant viruses were generously supplied by Prof. Sunwen Chou (Research Service, Department of Veterans Affairs Medical Center, Portland, OR, USA). The ACV-R Clone 4, AD-169 CDV-R clone 5, AD-169 GCV-R clone 4, AD-169 PFA-R clone C, AD-169 PMEDAP-R clone 4, and AD-169 HPMPA-R clone 6 mutant viruses were isolated in vitro in our laboratory under pressure of, respectively, ACV, CDV, GCV, PFA, PMEDAP, and HPMPA. Virus clones were isolated from the virus culture stock by serial dilution on HEL fibroblasts and the purity of the viral mutants was checked by deep-sequencing.

All viral mutants as well as the HEL cells were screened for the presence of mycoplasma and were used only when they were negative.

### 2.4. Cytopathic Effect (CPE) Reduction Assays

Confluent monolayers of HEL cells (grown in 96-well plates) were inoculated with the different viral clones at an input of 100-fold the 50% cell culture infective dose (CCID_50_). The medium was replaced 2 h post-infection, after which serial dilutions of antiviral compounds were added in duplicate. At 7–8 days post-infection, viral cytopathic effect (CPE) was recorded under microscopic examination after ethanol fixation and Giemsa staining of the 96-well plates. Scores were attributed to each well on a scale from 0 to 5, with a score of 0 corresponding to no CPE and a score of 5 corresponding to 100% CPE. The 50% effective concentration (EC_50_) was defined as the concentration of a compound required to reduce the viral CPE by 50%. Fold resistance (Fold-R) was determined for each drug as the ratio EC_50_ mutant virus/EC_50_ wild-type virus.

### 2.5. Genotyping by Sanger Sequencing

DNA from virus-infected cell cultures was extracted with a Qiamp^®^ DNA Blood Mini Kit (Qiagen, Venlo, The Netherlands). Three (UL97 protein kinase, PK) and five (UL54, DNA polymerase, DNA pol) overlapping primer sets were used. Primers with 5′-end flanked with universal M13 sequences (to allow sequencing of the amplicons with universal M13-primers), covering the regions of the viral genes where drug-R mutations map (nucleotides 990–2124 and 800–3260, respectively) were designed. For the UL56 terminase subunit, six overlapping primer sets, 5′-end flanked with universal M13 sequences, covering the entire viral gene were designed. PCR amplifications were carried out using the Faststart High Fidelity PCR System kit (Roche) and amplification products were purified with the QIAquick PCR Purification Kit (Qiagen). All Sanger sequencing assays were performed bidirectionally using M13 primers. Gene amplicons were directly sequenced with the BigDye Terminator v3.1 sequencing kit (Thermo Fisher Scientific). Sequencing reactions were purified using the Applied Biosystems™ BigDye XTerminator™ Purification Kit (Thermo Fisher Scientific) and analyzed with the automated sequencer ABI 3730 genetic analyzer (Applied Biosystems, Merelbeke, Belgium). The sequencing results were computer assembled and compared with UL97, UL54, and UL56 sequences from the reference AD-169 CMV strain (Genbank accession number X17403) using the software SeqScape, version 2.7 (Applied Biosystems).

### 2.6. Dual-Infection Competition Assays

Confluent HEL fibroblasts in 24-well plates were infected with 100 CCID_50_ of a single virus, a 50:50 ratio of wild-type and mutant viruses, or a 50:50 ratio of two mutants. DNA was extracted from the viral inoculum at the time of infection (Day 0). At two hours post-infection, viral inoculum was removed and 1.0 mL fresh medium containing cidofovir (CDV, 4 and 1 µM), ganciclovir (GCV, 4 and 1 µM), foscarnet (PFA, 100 and 40 µM), adefovir (PMEA, 100 and 40 µM or 40 and 10 µM), acyclovir (ACV, 100 and 40 µM), letermovir (LMV, 1, 0.4, 0.1, 0.04, and 0.01 µM) or no antiviral was added. The selected antiviral concentrations allowed limited replication of both wild-type and mutant viruses. Plates were frozen 7 days post-infection. After thawing, supernatants were harvested and centrifuged at 1800 rpm for 10 min followed by DNA extraction. Frequencies of viral populations at Days 0 and 7 were determined by NGS.

### 2.7. Detection of Viral Variants by Next-Generation Sequencing (NGS)

The Platinum^TM^ Superfi II PCR Mastermix (Thermo Fisher Scientific) was used to amplify ∼2 kb (UL56) and ∼4 kb (UL54) targets. PCR products were purified [QIAquick PCR Purification Kit (Qiagen)], quantified [Qubit dsDNA HS Assay Kit on the Qubit^®^ 2.0 fluorometer (Thermo Fisher Scientific)] and handled to prepare DNA libraries (one library for each sample) using 1 ng DNA with the Nextera XT DNA Sample Preparation Kit and the Nextera XT DNA Sample Preparation Index Kit (Illumina Netherlands, Eindhoven, The Netherlands)]. Resulting dsDNA products were purified using Agencourt^®^ AMPure^®^ XP beads. Quality, fragment length distribution, and concentration of the libraries were assessed (Qubit^®^ fluorometer and Qubit^®^dsDNA HS Assay Kit). All NGS libraries were diluted to a 2 nM concentration and equal volumes of 5 μL of each library were pooled. After normalization, spiking of the libraries with a 5% PhiX Control v3 library (12.5 pM; Illumina) was carried out. The library pool was sequenced using paired-end (2 × 150 bp) reads on the Miseq v.2 system (Illumina). Primary data analysis was done using the “Sequencing Analysis Viewer” software v1.8.11 (Illumina) and secondary data analysis was performed with the CLC bio Genomics Workbench version 8.0.2 software (Qiagen). Mapping was performed with local alignment of the reads to the reference sequences and variants were called with the Quality-based variant detection tool to detect minor viral variants. Deep-sequencing data were confirmed by analyzing two independent PCR target amplifications for each viral gene.

## 3. Results

### 3.1. Genotypic Characterization of CMV Wild-Type (WT) and Mutant Viruses

The wild-type and CMV mutants were analyzed by NGS using the MiSeq Illumina platform to determine the purity of the mutations associated with drug-R. The frequency of the viral variants in the DNA polymerase (DNA pol, UL54) and/or in the terminase subunit UL56 were determined in duplicate. In some cases, a second or third round of clone isolation was necessary to obtain pure viral populations. Only viral clones showing a variant frequency > 99% were used for the subsequent experiments (Supplementary Table S1).

### 3.2. Phenotypic Characterization of the Different Drug-R Viruses

The results of the drug-susceptibility profile in HEL cells, determined by cytopathic effect (CPE) reduction assay, are summarized in Table 1, Table 2 and Table 3. Different DNA pol mutants selected under pressure of nucleoside [ganciclovir (GCV), or acyclovir (ACV)], nucleotide [cidofovir (CDV, HPMPC), HPMPA, and PMEDAP] or pyrophosphate [foscarnet (PFA)] analogues (denoted, respectively, GCV-R, ACV-R, CDV-R, HPMPA-R, PMEDAP-R, and PFA-R) were included in the study. Most of the CMV DNA pol mutants showed specific changes in the CMV DNA polymerase known to be associated with drug resistance, including A987G (CDV-R), K513N (GCV-R), and V715M (PFA-R). A novel DNA pol change H729Y was identified in the ACV-R mutant while the HPMPA-R viruses harboured a known drug-R substitution (F412L) and a novel change of unknown significance (Q783H). Four different letermovir resistance (LMV-R) viruses bearing changes at codon 325 (C325F/Y/W) or 236 (V236M) in the UL56 terminase subunit were also characterized phenotypically.

Specific patterns of drug-susceptibility/resistance profiles for the various DNA pol inhibitors were found for the DNA pol mutant viruses, while no changes in LMV EC_50_ values were noted. The DNA pol mutants bearing the A987G or K513N mutations clearly showed cross-resistance to CDV, GCV, and HPMPA. The latter drug belongs, together with CDV, to the group of 3-hydroxy-2-phosphonylmethoxypropyl (HPMP) derivatives. Both CDV (HPMPC) and HPMPA share the same basic structural component (HPMP), but the base they are attached to is different, i.e., adenine for HPMPA and cytosine for HPMPC. The CDV-R (A987G) and GCV-R (K513N) viruses remained sensitive to the nucleoside analogue ACV, the pyrophosphate analogue PFA, and the terminase inhibitor LMV, as well as to another class of acyclic nucleoside phosphonates (ANPs), the PME derivatives, represented by PMEA (ADV) and PMEDAP, which are characterized by a 2-(phosphonomethoxy)ethyl backbone. Interestingly, the K513N was linked to hypersensitivity to the PME derivatives and to ACV, with fold-R in the range of 0.2–0.33. The mutant selected under HPMPA pressure bearing the known drug-R substitution F412L [41,42] and a novel change of unknown significance Q783H in the DNA pol also showed a higher susceptibility to ADV (PMEA), PMEDAP, and ACV than the wild-type virus and also decreased sensitivity to the HPMP derivatives and GCV. The F412L + Q783H mutant had no changes in the EC_50_ values for LMV and PFA.

Mutants selected under PFA (V715M), ACV (H729Y) or PMEDAP (L773V) presented a comparable drug-susceptibility profile: sensitivity to the HPMP derivatives HPMPA and HCMPC (CDV), to the nucleoside analogue GCV, and to the terminase inhibitor LMV, and cross-resistance to PFA, ACV, and the PME derivatives PMEA (ADV) and PMEDAP. Although the L773V change was previously linked to low levels resistance to CDV and GCV and intermediate levels of resistance to PFA [43], we did not detect resistance to CDV and GCV under our experimental conditions.

While all UL54 DNA pol mutants remained sensitive to LMV, the LMV-R viruses showed very high levels of resistance to LMV while preserving their sensitivity to the diverse classes of CMV DNA pol inhibitors.

### 3.3. Fitness of CMV UL56 Terminase Mutants in Competition with Wild-Type (WT) Virus

**Figure 1:**
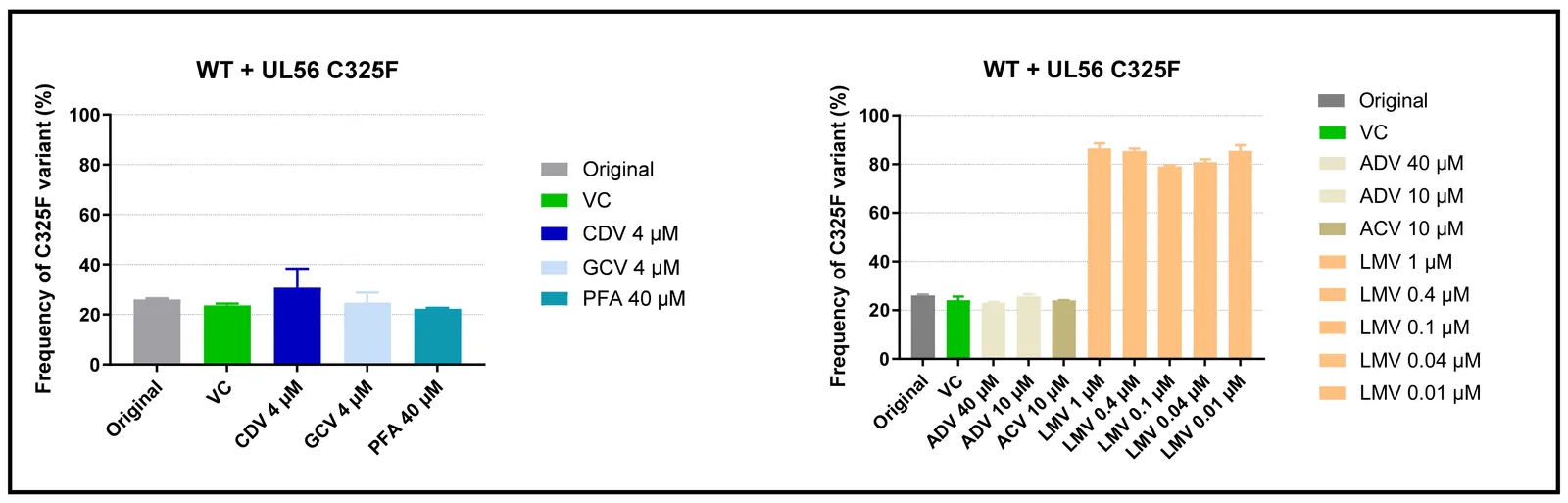
**Competition assays of wild-type (WT) virus with the UL56 C325F mutant.** HEL cells were co-infected with the reference AD-169 WT strain and mutant virus stocks mixed at a calculated 50:50 ratio. Growth competition was evaluated without drug pressure (virus control, VC) and under pressure of cidofovir (CDV, 4 µM), ganciclovir (GCV, 4 µM), foscarnet (PFA, 40 µM), adefovir (ADV, PMEA, 40 and 10 µM), acyclovir (ACV, 10 µM), or letermovir (LMV, 1, 0.4, 0.1, 0.04, and 0.01 µM). The frequencies of the mutant virus were quantified at the time of infection (day 0, original) and at 7 days post-infection by deep sequencing. Data are mean with standard deviations from two duplicates.

The replication capacity of the different UL56 mutants relative to wild-type virus was evaluated by dual-infection competition assays in the absence of antiviral drugs and under antiviral pressure of CDV, GCV, PFA, ADV, ACV, or LMV. The UL56 C325F terminase mutant had a similar fitness to the wild-type virus in the absence of antivirals or under pressure of the different DNA pol inhibitors (CDV, GCV, PFA, and ADV) as shown by the maintenance of the UL56 C325F variant frequency when comparing the NGS data at the time of infection (day 0, original) and after 7 days of growth without antivirals (virus control, VC), or under CDV, GCV, PFA, or ADV (Figure 1). A considerable gain in fitness of the UL56 mutant virus was found under pressure of LMV, with the C325F variant being quantified at a frequency of ~80% after 7 days of growth under different LMV concentrations vs. ~20% frequency at the time of infection.

**Figure 2:**
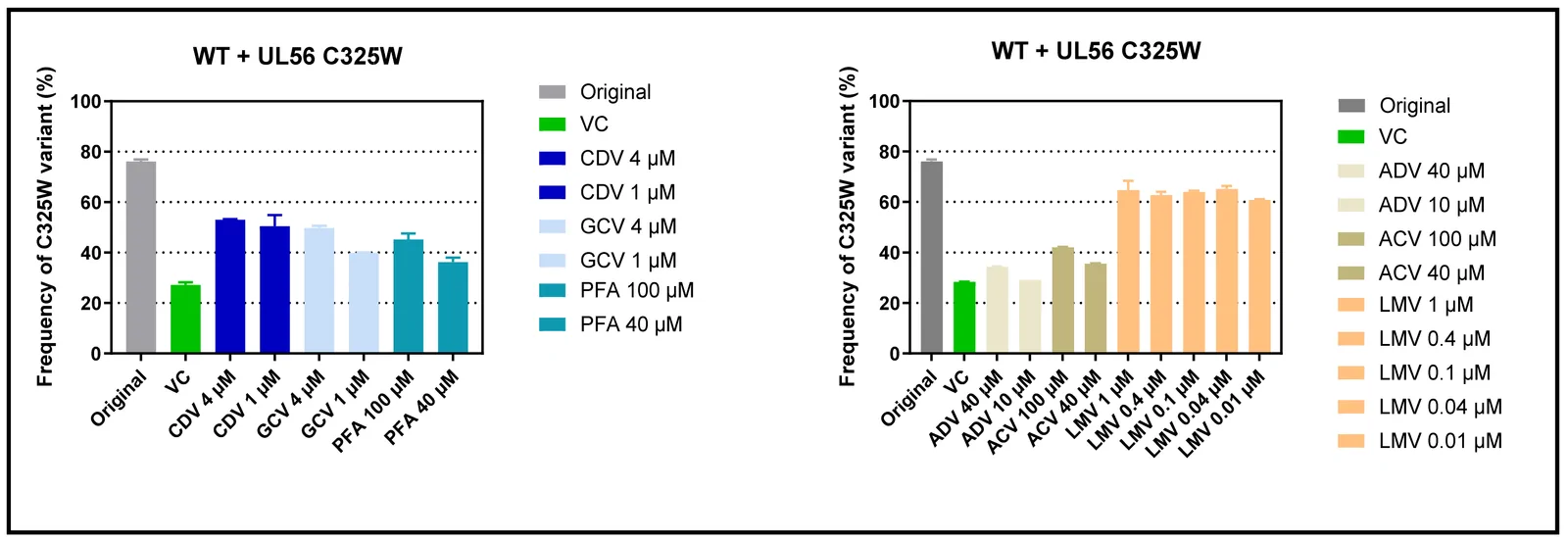
**Competition assays of wild-type (WT) virus with the UL56 C325W mutant.** HEL cells were co-infected with the reference AD-169 WT strain and mutant virus stocks mixed at a calculated 50:50 ratio. Growth competition was evaluated without drug pressure (virus control, VC) and under pressure of cidofovir (CDV, 4 and 1 µM), ganciclovir (GCV, 4 and 1 µM), foscarnet (PFA, 100 and 40 µM), adefovir (ADV, PMEA, 40 and 10 µM), acyclovir (ACV, 100 and 40 µM), or letermovir (LMV, 1, 0.4, 0.1, 0.04, and 0.01 µM). The frequencies of the mutant virus were quantified at the time of infection (day 0, original) and at 7 days post-infection by deep sequencing. Data are mean with standard deviations from two duplicates.

In contrast to the UL56 C325F mutant, the UL56 C325W mutant had a pronounced reduction in virus fitness when grown in competition with the wild-type virus in the absence of antivirals and in the presence of different DNA pol inhibitors (Figure 2). Even in the presence of LMV, the C325W mutant had a partial recovery of its replicative capacity since it was still impaired compared to the wild-type virus.

**Figure 3:**
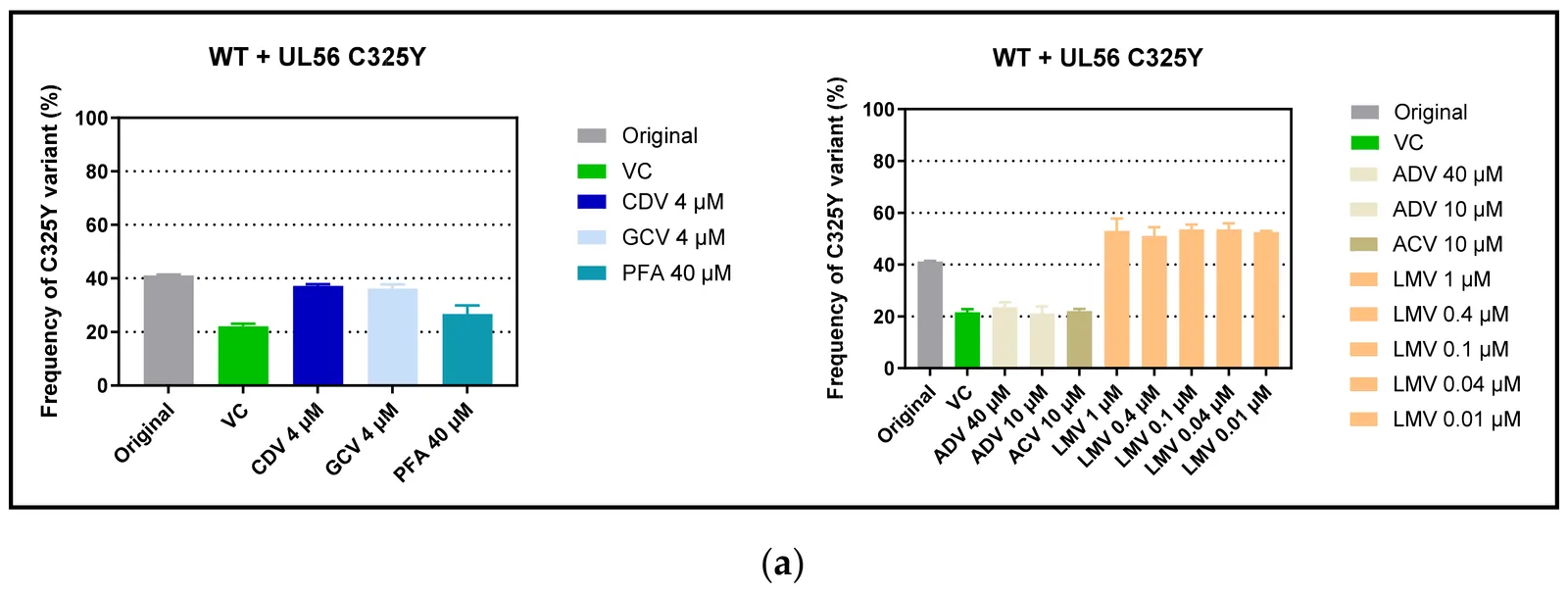

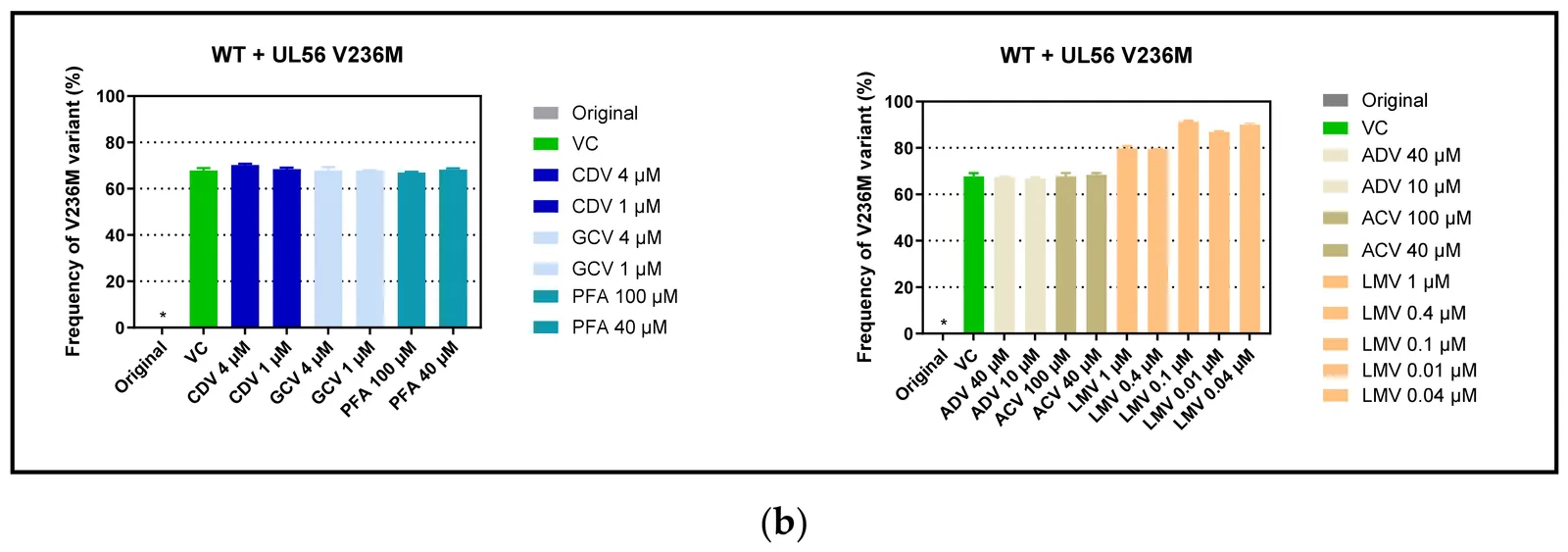
**Competition assays of wild-type (WT) virus with the UL56 C325Y (a) or the UL56 V236M (b) mutant viruses.** HEL cells were co-infected with the reference AD-169 wild-type (WT) strain and mutant virus stocks mixed at a calculated 50:50 ratio. Growth competition was evaluated without drug pressure (virus control, VC) and under pressure of cidofovir (CDV, 4 and 1 µM), ganciclovir (GCV, 4 and 1 µM), foscarnet (PFA, 100 and 40 µM), adefovir (ADV, PMEA, 40 and 10 µM), acyclovir (ACV, 100 and 40 µM), or letermovir (LMV, 1, 0.4, 0.1, 0.04, and 0.01 µM). The frequencies of the mutant viruses were quantified at the time of infection (day 0, original) and at 7 days post-infection by deep sequencing. Data are mean with standard deviations from two duplicates. (*) Data at the time of infection (original) not available.

The UL56 C325Y terminase mutant also had a reduction in replication capacity in the absence of antivirals and in the presence of the DNA pol inhibitors PFA, ACV and ADV with a negligible reduction in viral fitness in the presence of CDV and GCV (Figure 3a). When grown under LMV, the C325Y could slightly overgrow the wild-type virus, though to a much lower extent compared to the C325F mutant.

Regarding the V236M mutant, since the data for the original sample (virus inoculum at day 0) are missing, the fitness of this mutant relative to the wild-type could not be determined. Nevertheless, it can be concluded that none of the DNA pol inhibitors affected the frequency of the V236M variants when comparing the frequencies after 7 days of growth of this mutant without drugs and with the different DNA pol inhibitors. In contrast, the V236M variant after 7 days of growth under LMV was found at an increased frequency relative to the virus control (no antivirals) (Figure 3b).

### 3.4. Fitness of Two CMV UL56 Terminase Mutants Grown in Competition

The replication capacity of the different UL56 mutants was evaluated by dual-infection competition assays using diverse combinations of UL56 mutants in the absence of antiviral drugs and under antiviral pressure of CDV, GCV, PFA, ADV, ACV, or LMV. In these experiments the frequencies for the different UL56 mutants are reported as they could be detected simultaneously by targeted NGS of the UL56 gene.

**Figure 4:**
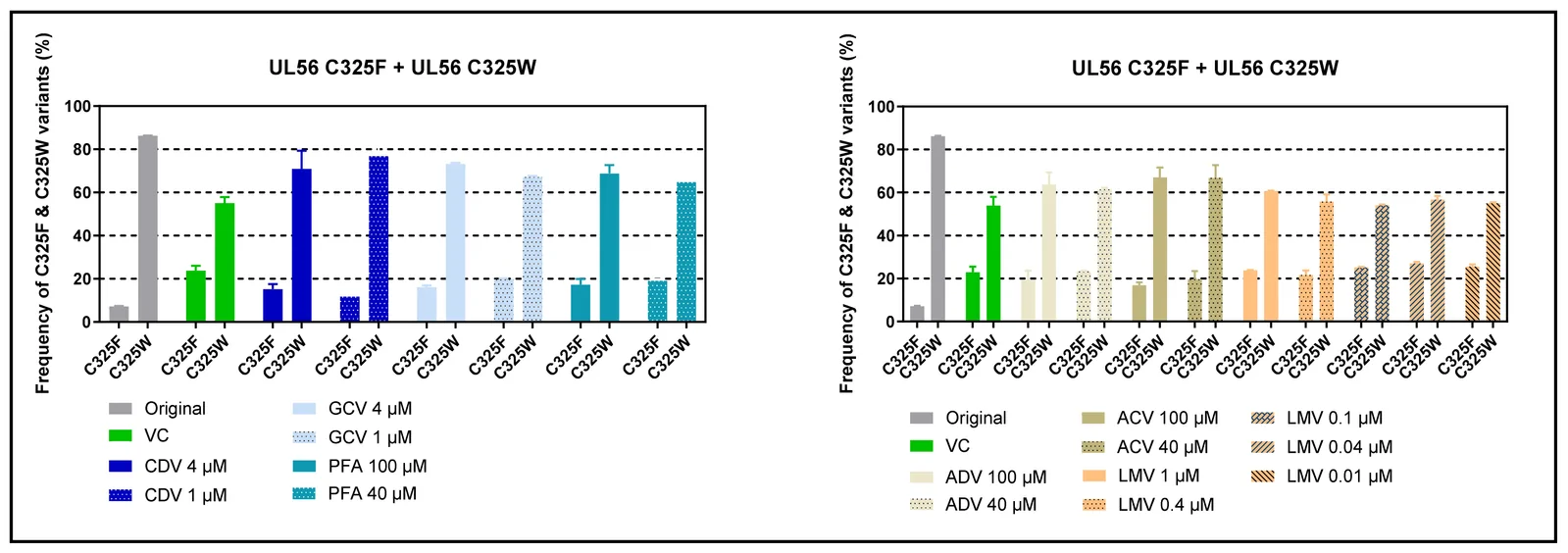
**Competition assays of the UL56 C325F and C325W mutants.** HEL cells were co-infected with the UL56 C325F and the UL56 C325W mutant stocks mixed at a calculated 50:50 ratio. Growth competition was evaluated without drug pressure (virus control, VC) and under pressure of cidofovir (CDV, 4 and 1 µM), ganciclovir (GCV, 4 and 1 µM), foscarnet (PFA, 100 and 40 µM), adefovir (ADV, PMEA, 100 and 40 µM), acyclovir (ACV, 100 and 40 µM), or letermovir (LMV, 1, 0.4, 0.1, 0.04, and 0.01 µM). The frequencies of the mutant virus were quantified at the time of infection (day 0, original) and at 7 days post-infection by deep sequencing. Data are mean with standard deviations from two duplicates.

The results of the C325F + C325W dual-infection competition assay showed that although the starting ratio of the C325F mutant to the C325W mutant was low, the C325F mutant had a superior replication capacity compared to the UL56 C325W mutant when grown either in the absence of drugs or in the presence of anti-CMV drugs (different DNA pol inhibitors and LMV) (Figure 4). This points to a higher replication capacity of the C325F over the C325W mutant independent of the drug selective pressure, which would favour the selection of the C325F mutant over the C325W under (non)-selective conditions.

**Figure 5:**
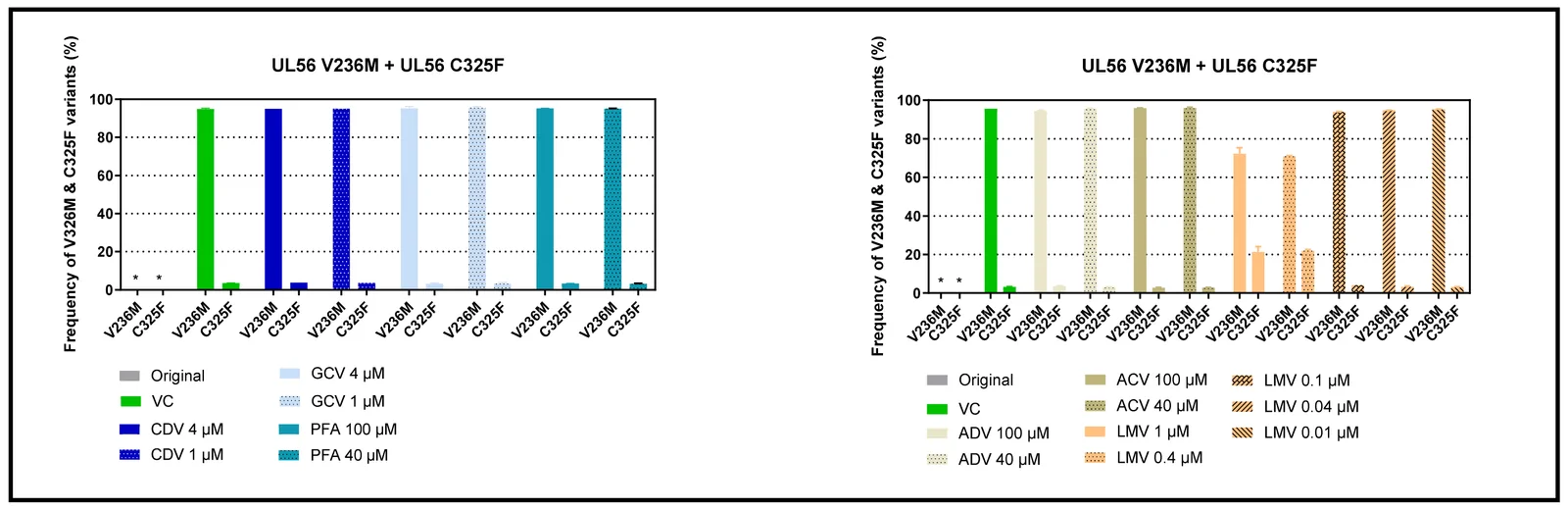
**Competition assays of the UL56 V236M and C325F mutants.** HEL cells were co-infected with the UL56 V236M and the UL56 C325F mutant stocks mixed at a calculated 50:50 ratio. Growth competition was evaluated without drug pressure (virus control, VC) and under pressure of cidofovir (CDV, 4 and 1 µM), ganciclovir (GCV, 4 and 1 µM), foscarnet (PFA, 100 and 40 µM), adefovir (ADV, PMEA, 100 and 40 µM), acyclovir (ACV, 100 and 40 µM), or letermovir (LMV, 1, 0.4, 0.1, 0.04, and 0.01 µM). The frequencies of the mutant viruses at 7 days post-infection were quantified by deep sequencing. Data are mean with standard deviations from two duplicates. (*) Data at the time of infection (original) not available.

The replication capacity of the C325F mutant was also evaluated when competing with the V236M mutant (Figure 5). Because data from the original sample (time 0) are missing, we can only compare the replication of the V236M and of the C325F mutants with drugs vs. no antivirals after 7 days of growth. The growth of the M236V and C325F UL56 mutants was equivalent in the presence of DNA pol inhibitors or without drugs. However, at 1 µM and 0.4 µM LMV concentrations, the replication capacity of the C325F was superior to that of the V236M [the C325F mutant was found at a higher frequency than the V236M mutant when comparing the results for 1 µM and 0.4 µM LMV to the virus control (no drug pressure)]. These findings support the fact that the C325F is the most frequent UL56 change associated with LMV resistance in the clinic.

**Figure 6:**
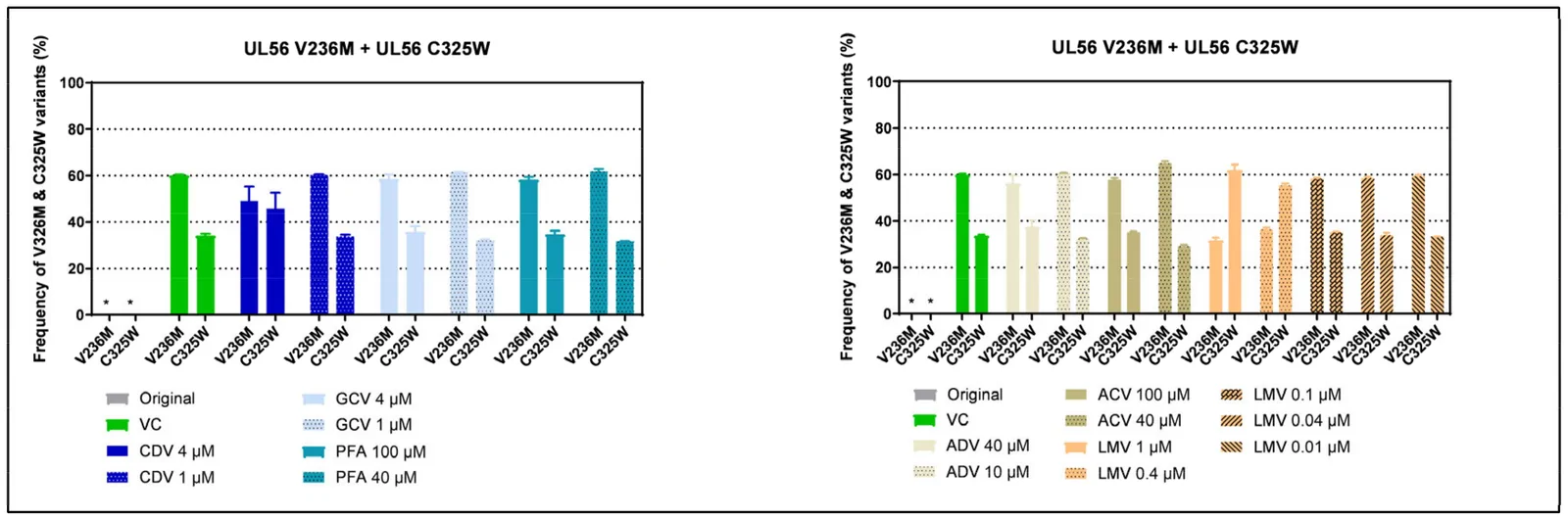
Competition assays of the UL56 C325W and V236M mutants. HEL cells were co-infected with the UL56 C325F and the UL56 V236M mutant stocks mixed at a calculated 50:50 ratio. Growth competition was evaluated without drug pressure (virus control, VC) and under pressure of cidofovir (CDV, 4 and 1 µM), ganciclovir (GCV, 4 and 1 µM), foscarnet (PFA, 100 and 40 µM), adefovir (ADV, PMEA, 40 and 10 µM), acyclovir (ACV, 100 and 40 µM), or letermovir (LMV, 1, 0.4, 0.1, 0.04, and 0.01 µM). The frequencies of the mutant virus at at 7 days post-infection were quantified by deep sequencing. Data are mean with standard deviations from two duplicates. (*) Data at the time of infection (original) not available.

When the V236M mutant and the C325W mutant were grown in competition, their fitness was not affected by the presence of anti-CMV drugs, expect for LMV (Figure 6). At 1 µM and 0.4 µM LMV concentrations, the replication capacity of the C325W was superior to that of the V236M.

### 3.5. Fitness of UL54 DNA Polymerase (DNA Pol) Mutants in Competition with Wild-Type (WT) Virus

**Figure 7:**
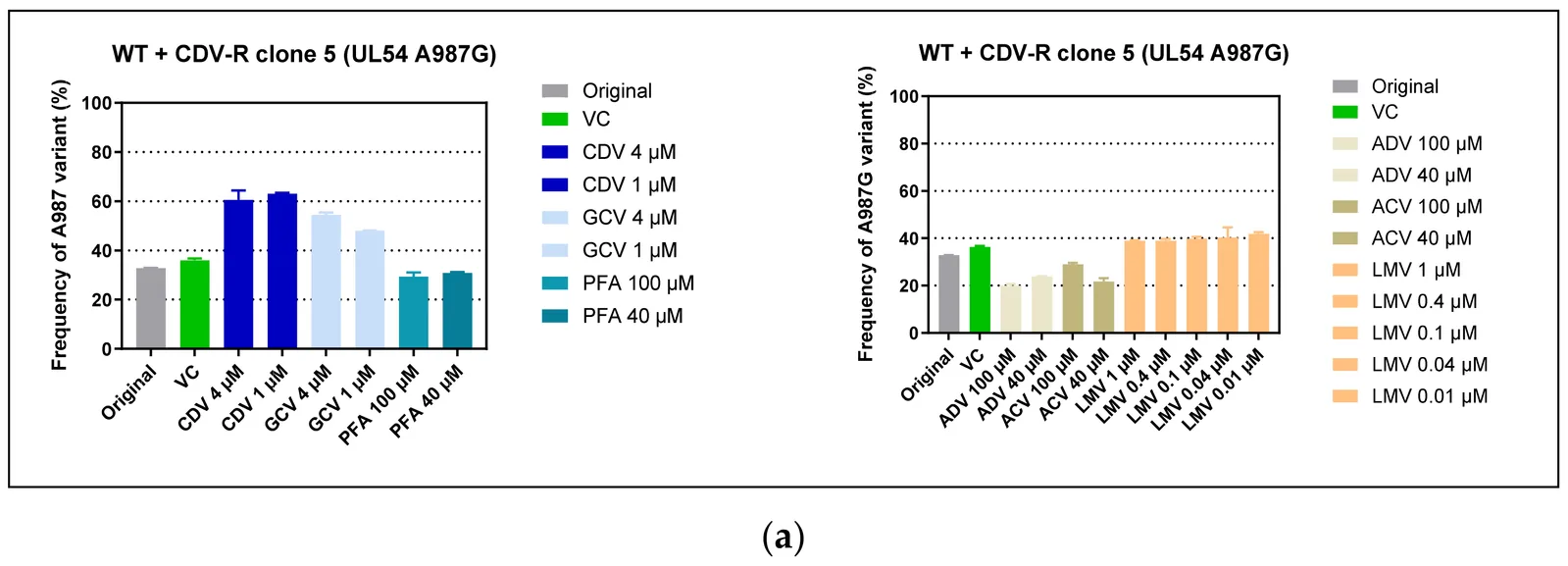

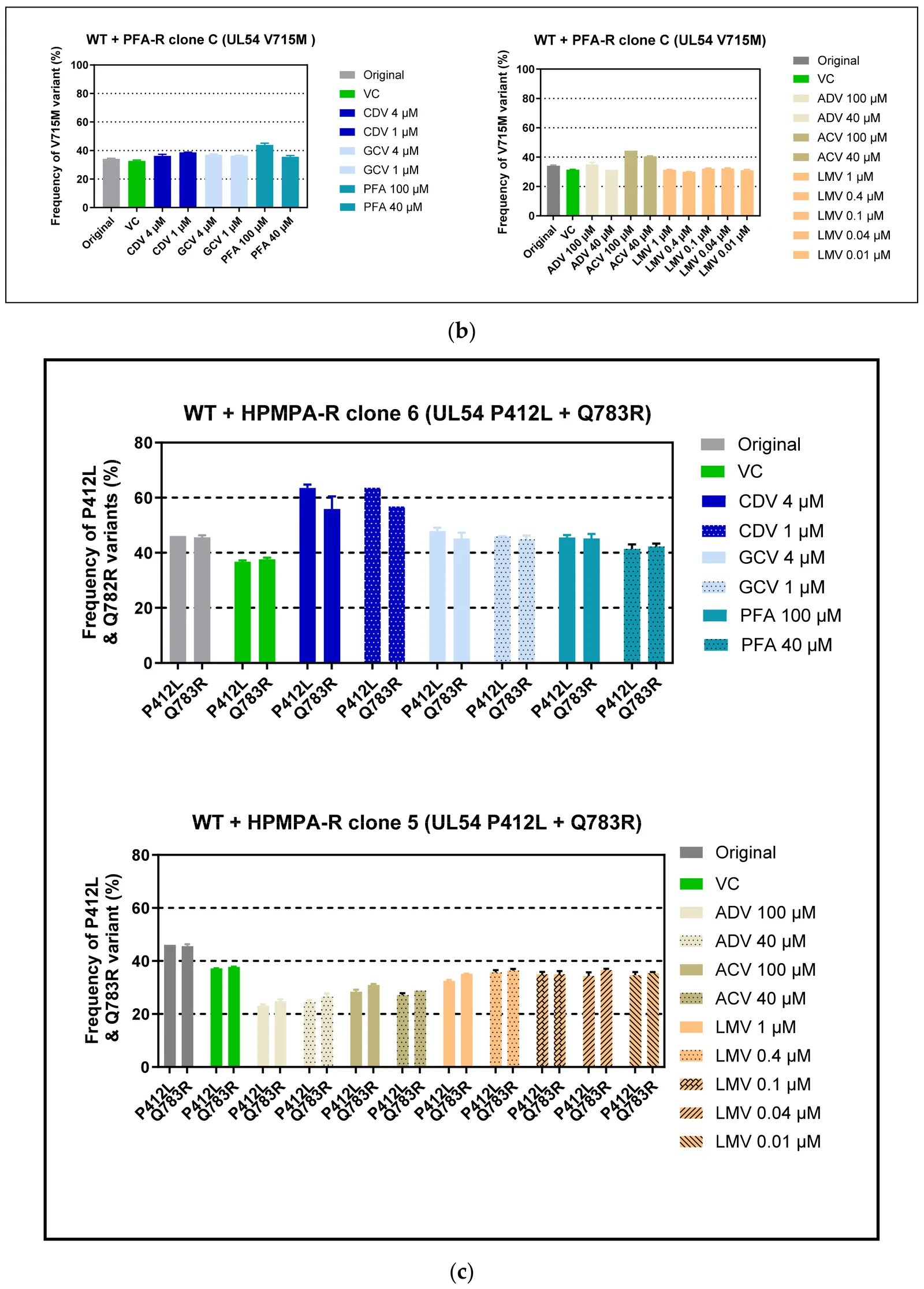
**Competition assays of wild-type (WT) virus with the UL54 DNA pol A987G (a), UL54 DNA pol V715M (b) or UL54 DNA pol F412L + Q783R (c) mutants.** HEL cells were co-infected with the reference AD-169 wild-type (WT) strain and mutant virus stocks mixed at a calculated 50:50 ratio. Growth competition was evaluated without drug pressure (virus control, VC) and under pressure of cidofovir (CDV, 4 and 1 µM), ganciclovir (GCV, 4 and 1 µM), foscarnet (PFA, 100 and 40 µM), adefovir (ADV, PMEA, 100 and 40 µM), acyclovir (ACV, 100 and 40 µM), and letermovir (LMV, 1, 0.4, 0.1, 0.04, and 0.01 µM). The frequencies of the mutant virus were quantified at the time of infection (day 0, original) and at 7 days post-infection by deep sequencing. Data are mean with standard deviations from two duplicates.

Next, we evaluated the replication capacity of DNA pol mutants in competition with the reference CMV AD-169 strain. The UL54 A987V mutant gained fitness in the presence of CDV and GCV, in line with the GCV-R and CDV-R phenotype associated with this mutation (Figure 7a). Without antiviral pressure and in the presence of LMV, this viral mutant proved equally fitted as the wild-type.

When the UL54 DNA pol V715M was grown in competition with the wild-type virus, the mutant virus was equally fitted as the wild-type, except when grown under PFA at 100 µM and under ACV at 40 µM and 100 µM, in line with the resistance to these drugs conferred by the DNA pol V715M change (Figure 7b). Although this mutant displayed resistance to ADV, the mutant virus was not able to overgrow the wild-type virus under ADV pressure, which may be explained by the low level of resistance to this drug (about 3-folds).

For the HPMPA-R virus, bearing the F412L & Q783R UL54 changes in the DNA pol, the frequency of both mutations could be measured independently by NGS as they map to the same viral gene. Because they are found at an equivalent frequency, it can be assumed that they are in the same backbone and are not two independent mutants, which is in line with the fact that all experiments were done with viral clones, i.e., pure viral populations. The F412L & Q783R DNA pol mutant was less fitted than the wild-type virus when competing in the absence of antivirals or under pressure of ADV, ACV, or LMV (Figure 7c). The lowest decrease in replication capacity for this mutant was found with ACV and ADV, to which the UL54 F412L & Q783R mutant showed a clear hypersensitivity in the phenotypic assays. This double mutant proved equally fitted as the wild-type virus when grown in the presence of PFA or GCV and clearly gained fitness under CDV pressure, a drug to which the mutant was highly resistance.

In summary, DNA pol mutants showed a superior fitness when grown under pressure of drugs to which they showed resistance to and decrease fitness under pressure of drugs to which they displayed hypersensitivity.

### 3.6. Fitness of the UL56 C325F Terminase Mutant in Competition with a DNA Polymerase Mutant

**Figure 8:**
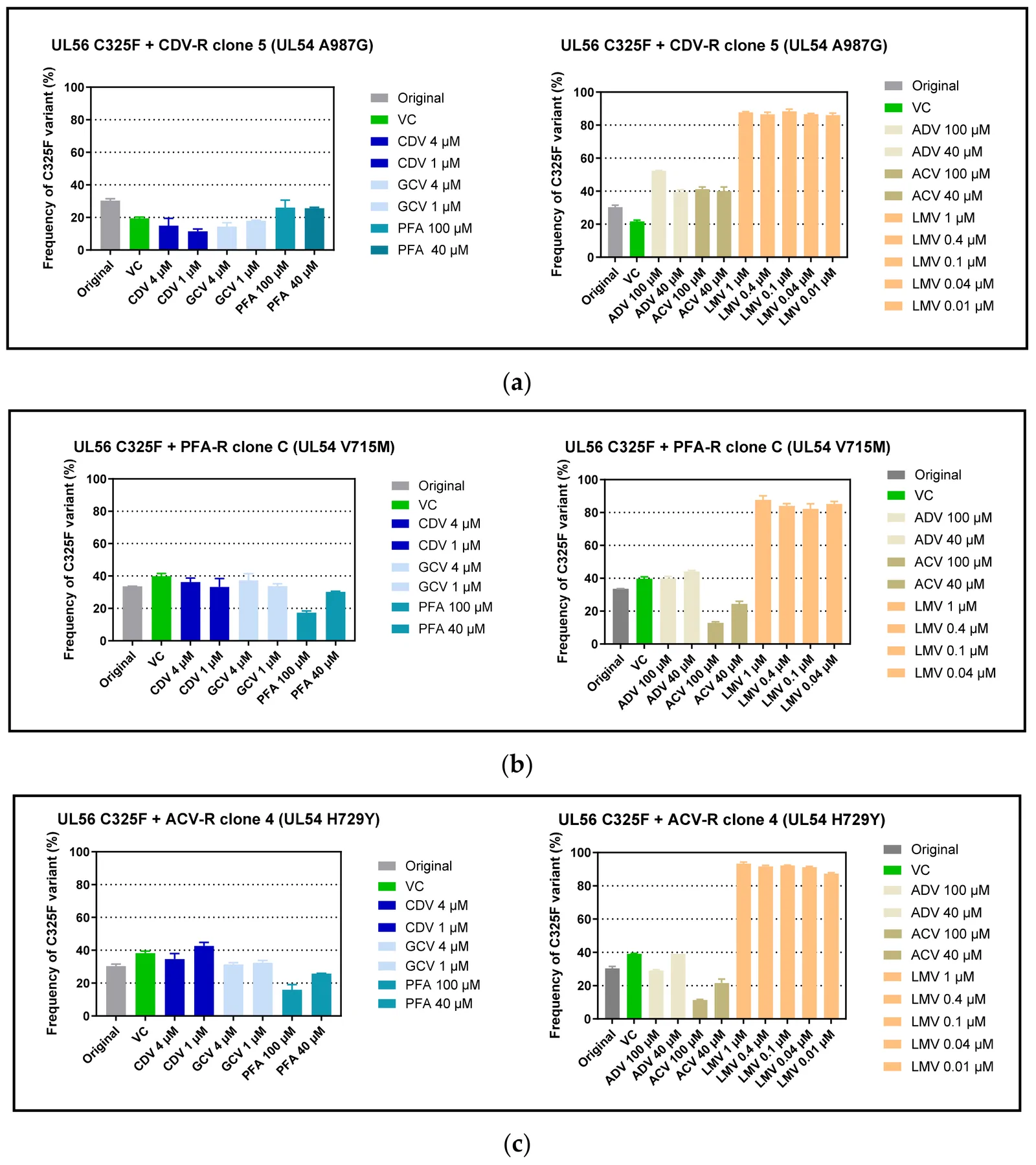
**Competition assays of the UL56 C325F terminase mutant virus with the UL54 DNA pol A987G (a), UL54 DNA pol V715M (b) or UL54 DNA pol H729Y (c) mutants.** HEL cells were co-infected with the reference AD-169 wild-type (WT) strain and mutant virus stocks mixed at a calculated 50:50 ratio. Growth competition was evaluated without drug pressure (virus control, VC) and under pressure of cidofovir (CDV, 4 and 1 µM), ganciclovir (GCV, 4 and 1 µM), foscarnet (PFA, 100 and 40 µM), adefovir (ADV, PMEA, 40 and 10 µM), acyclovir (ACV, 100 and 40 µM), and letermovir (LMV, 1, 0.4, 0.1, 0.04, and 0.01 µM). The frequencies of the mutant virus were quantified at the time of infection (day 0, original) and at 7 days post-infection by deep sequencing. Data are mean with standard deviations from two duplicates.

The replication capacity of the UL56 C325F terminase mutant (showing high levels of LMV-R) was investigated when grown in competition with some of the DNA pol mutants (Figure 8). The UL56 C325F virus was less fitted than the UL54 A987G mutant when grown in the absence of any antiviral drug or when grown under pressure of CDV, HPMPA, and GCV. The terminase mutant had a slight gain in replication capacity under PFA and ADV but a substantial advantage under LMV (Figure 8a).

When the UL56 C325F mutant was grown in competition with either the UL54 V715M (Figure 8b) or the UL54 H729Y (Figure 8c) mutants, the terminase mutant proved equally fitted as these DNA pol mutants when grown without selective drug pressure or under pressure of CDV or GCV. However, the C325F mutant had reduced fitness under PFA and ACV as the V715M and H729Y DNA pol mutations conferred substantial resistance to both PFA and ACV, but superior replication under LMV.

Notably, the UL56 C325F mutant gained striking replicative capacity in the presence of LMV when grown in competition with either the UL54 A987G, V715M, or H729Y DNA pol mutants.

### 3.7. Fitness of the UL56 C325W Terminase Mutant in Competition with a DNA Polymerase Mutant

**Figure 9:**
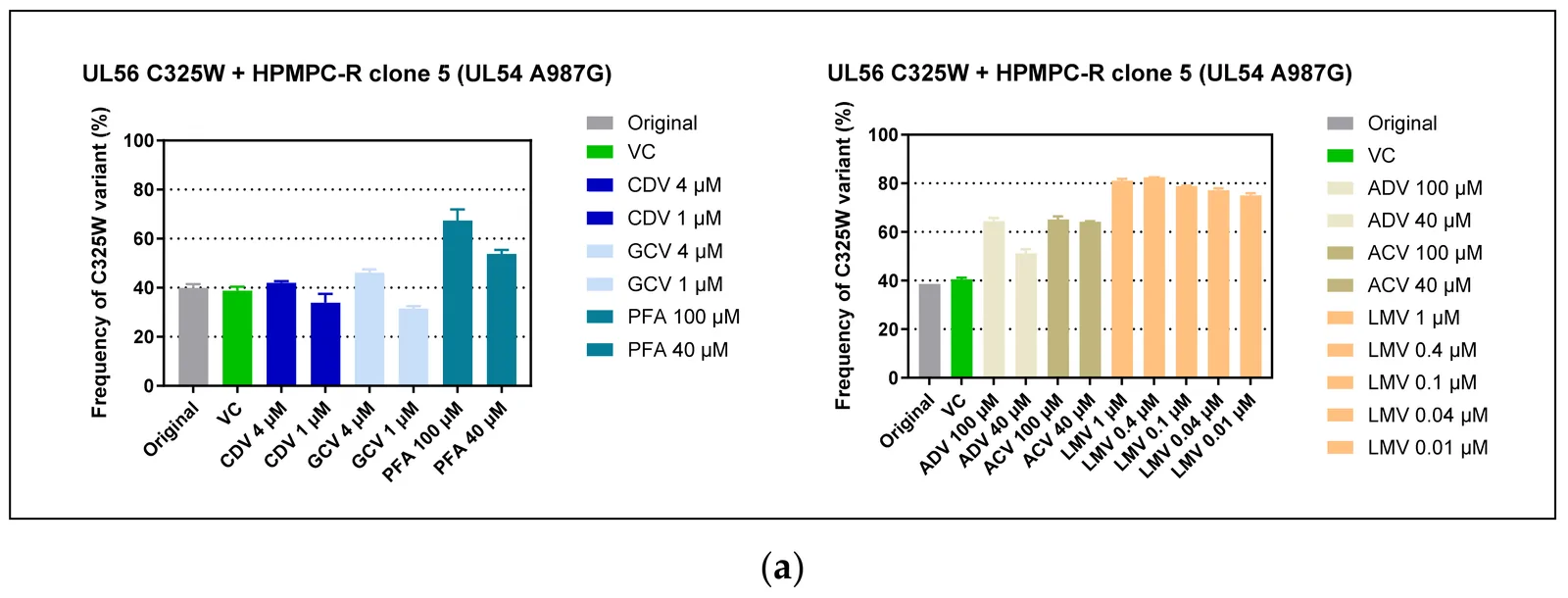

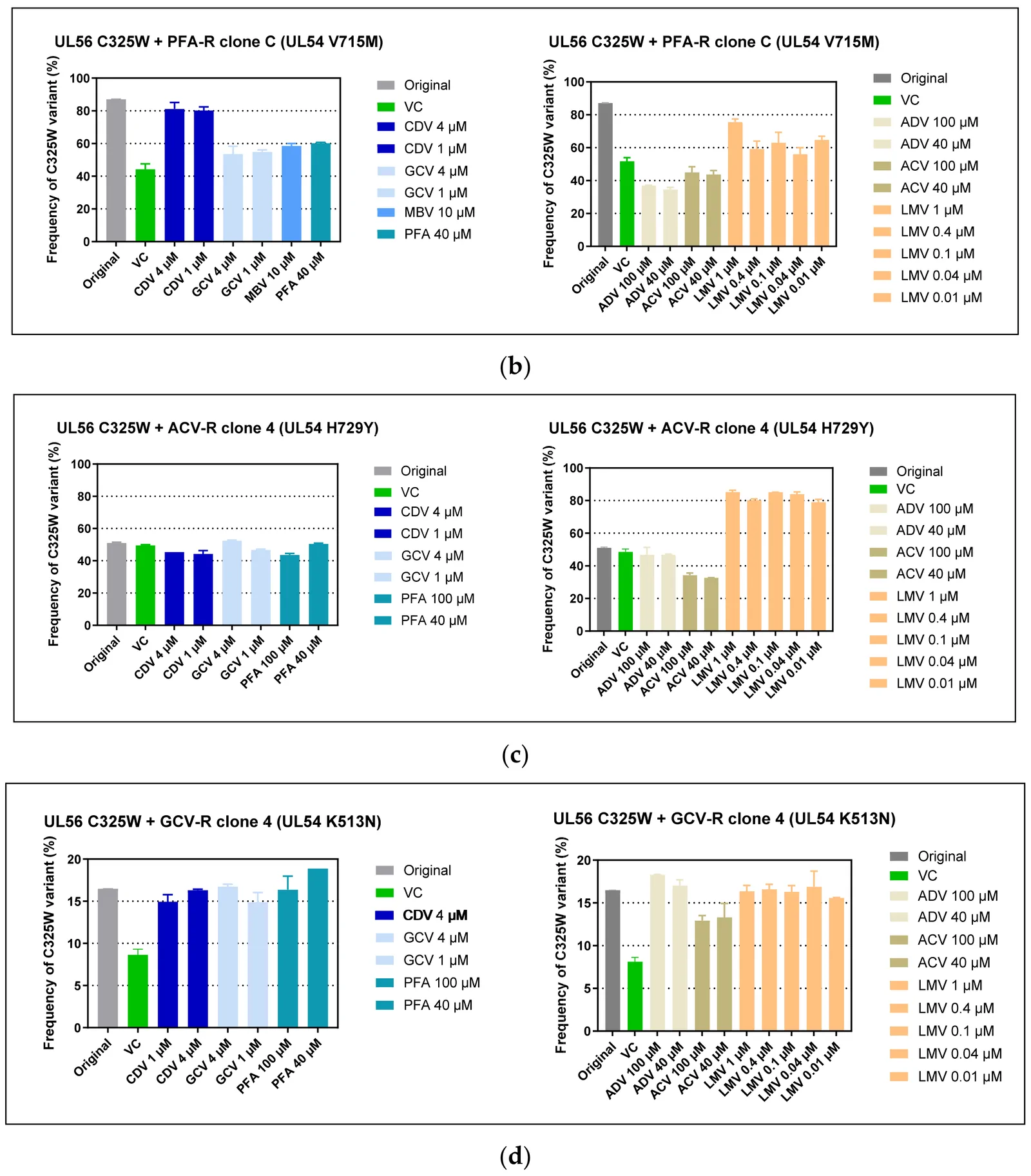
Competition assays of the UL56 C325W terminase mutant virus with the UL54 DNA pol A987G (**a**), UL54 DNA pol V715M (**b**), UL54 DNA pol H729Y (**c**), or UL54 DNA pol K513N (**d**) mutants. HEL cells were co-infected with the reference AD-169 wild-type (WT) strain and mutant virus stocks mixed at a calculated 50:50 ratio. Growth competition was evaluated without drug pressure (virus control, VC) and under pressure of cidofovir (CDV, 4 and 1 µM), ganciclovir (GCV, 4 and 1 µM), foscarnet (PFA, 100 and 40 µM), adefovir (PMEA, 100 and 40 µM), acyclovir (ACV, 100 and 40 µM), and letermovir (LMV, 1, 0.4, 0.1, 0.04, and 0.01 µM). The frequencies of the mutant virus were quantified at the time of infection (day 0, original) and at 7 days post-infection by deep sequencing. Data are mean with standard deviations from two duplicates.

The fitness of the C325W UL56 terminase mutant, also associated with high levels of resistance to LMV, was investigated in dual-infection competition assays with several DNA pol mutants (Figure 9). The UL56 C325W terminase mutant showed an important gain in fitness when grown in competition with the UL54 DNA pol A987V mutant in the presence of LMV (Figure 9a). An increase in replicative capacity was also found for this UL56 mutant in the presence of PFA, ADV, and ACV though to a lesser extent than under LMV pressure. These mutants were equally fitted in the absence of drug pressure or under CDV and GCV, the drugs to which the A987V DNA pol mutant showed resistance to.

In contrast, the UL56 C325W mutant had a reduced replication capacity compared to the UL54 V715M mutant when grown without antivirals or under pressure of GCV, PFA, ADV, or ACV, but not under CDV (Figure 9b). These results suggest that CDV is highly effective in inhibiting the replication of the V715M DNA pol mutant. In the presence of LMV, the UL56 C325W virus partially recovered its replication capacity but still had a reduced viral fitness compared to the V715 DNA pol mutant.

Although the replicative capacity of the UL54 H729Y mutant was not affected when competing with the UL56 C325W mutant without antivirals, its fitness was greatly reduced when grown under LMV since the C325W mutant overgrew the UL54 H729Y mutant virus (Figure 9c). Under ACV, the UL54 H729Y mutant had an advantage over the terminase mutant virus in agreement with the H729Y being associated with ACV-R. Without antiviral pressure or under CDV, GCV, or PFA, both mutants were maintained at a similar ratio after 7 days of competitive growth.

Interestingly, the fitness of the UL56 C325W mutant was considerably reduced when grown in competition with the UL54 K513N mutant in the absence of drug pressure (Figure 9d). However, under pressure with different antivirals, including LMV and the different DNA pol inhibitors, the C325W recovered its replication capacity.

### 3.8. Fitness of the UL56 C325Y Terminase Mutant in Competition with a DNA Polymerase Mutant

**Figure 10:**
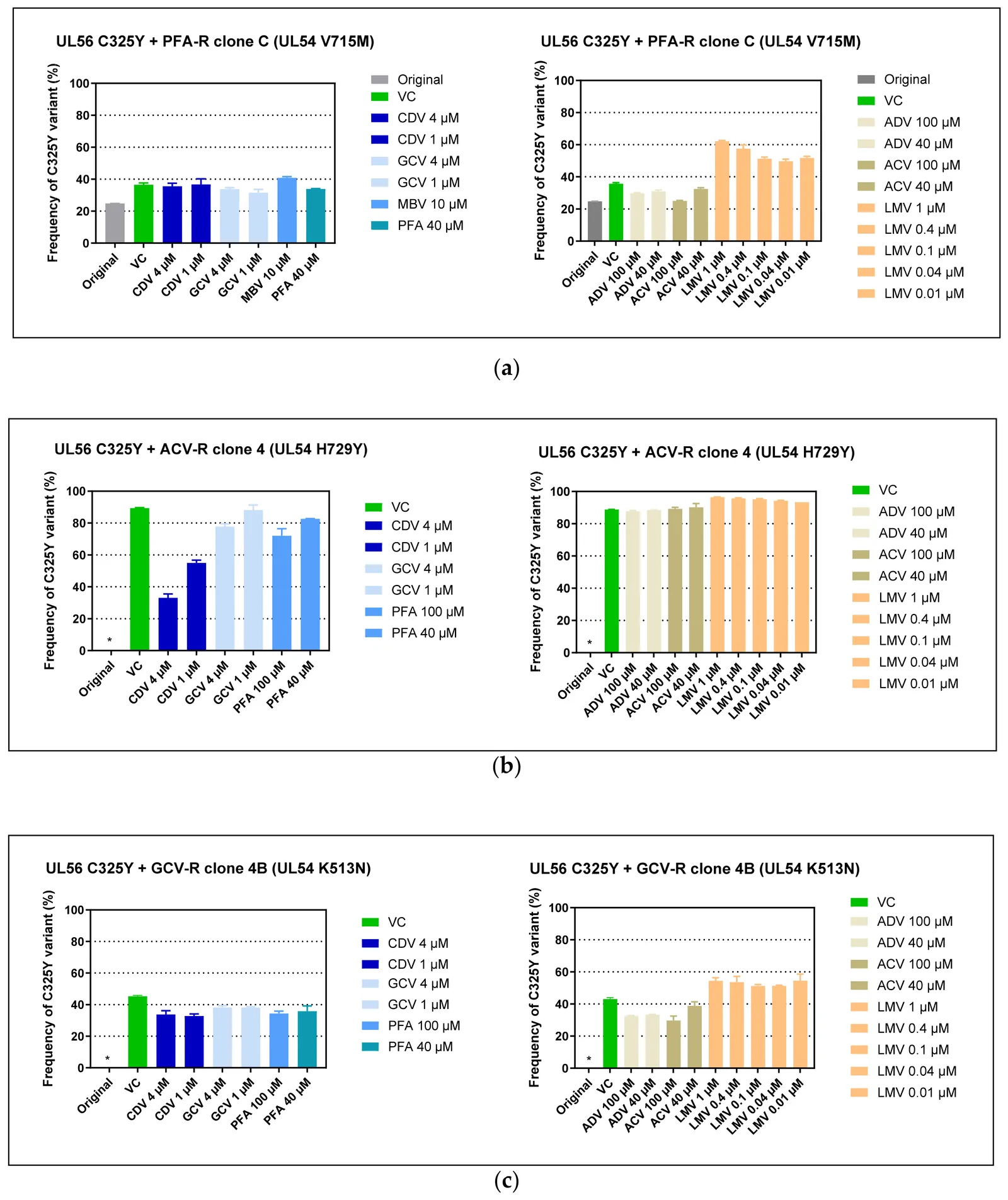
Competition assays of the UL56 C325Y terminase mutant virus with the UL54 V715M (**a**), UL54 DNA pol H729Y (**b**), or UL54 DNA pol K513N (**c**) mutants. HEL cells were co-infected with the reference AD-169 wild-type (WT) strain and mutant virus stocks mixed at a calculated 50:50 ratio. Growth competition was evaluated without drug pressure (virus control, VC) and under pressure of cidofovir (CDV, 4 and 1 µM), ganciclovir (GCV, 4 and 1 µM), foscarnet (PFA, 100 and 40 µM), adefovir (ADV, PMEA, 100 and 40 µM), acyclovir (ACV, 100 and 40 µM), and letermovir (LMV, 1, 0.4, 0.1, 0.04, and 0.01 µM). The frequencies of the mutant virus were quantified at the time of infection (day 0, original) and at 7 days post-infection (A) by deep sequencing. (*) The frequencies of the mutant viruses at the time of infection (day 0, original) are not available for the experiments presented in (**b**) and (**c**). Data are mean with standard deviations from two duplicates.

When the UL56 C325Y mutant virus grew in competition with the UL54 V715M mutant, the virus with a defect in the terminase showed a slightly better fitness in the presence of the DNA pol inhibitors and a substantial gain in replication capacity under LMV (Figure 10a).

When grown in competition with the DNA pol H729Y mutant, the UL56 C325Y terminase mutant showed a lower replicative capacity under CDV than when grown without antivirals or under ADV, ACV, or LMV (Figure 10b).

Under different DNA pol inhibitors, in particular ADV and ACV, the UL56 C325Y mutant in competition with the K513 DNA pol mutant grew at a lower capacity than in the absence of drugs (Figure 10c). However, this UL56 mutant grew slightly better under LMV than the UL54 K513N virus.

All in all, these results show that the UL56 C325F mutant had a much better fitness than the C325W/Y mutants under LMV when grown in competition with different DNA pol mutants.

## 4. Discussion

Regarding viral evolution and drug resistance, fitness cost, i.e., the ability of a virus to replicate and survive, needs to be considered. Mutations that allow CMV to evade LMV might come at a cost, impacting its overall fitness. This cost can manifest as a lower efficiency of viral replication in the absence of LMV, reduced infectivity leading to reduced transmissibility, or, eventually, hypersensitivity to other antiviral agents. The standard method that has been used to evaluate the replication capacity of CMV drug-R viruses is based on the analysis of growth curves of mutant viruses compared to wild-type virus in mono-infections [41,43,44,45,46]. By using this type of assays and in the absence of drug-pressure, recombinant viruses bearing mutations in the UL56 terminase subunit conferring low, medium, or high levels LMV-R, showed no or minimal growth impairment [24,47,48]. The majority of the clinically relevant LMV-R mutations cluster to the binding pocket region, within the amino acids 229–369 of the UL56 terminase subunit. Three of the most frequent LMV-R mutations, C325F/Y/W, result in a pronounced cavity reduction, with LMV being pushed out of the binding pocket [19]. Interestingly, the R246C UL56 mutant, detected in 2/80 transplant recipients before LMV therapy in refractory CMV patients, showed advanced viral fitness and sensitivity to GCV, CDV, PFA, MBV (maribavir), and LMV [49].

In this study, by performing dual-infection competition assays we showed that competition with wild-type and drug pressure can significantly affect the fitness of drug-R CMV mutants. Several important conclusions can be drawn from our work. The UL56 C325F terminase mutant showed the highest replication capacity among the different investigated terminase mutants when grown in competition with the wild-type virus under LMV pressure. Further, the UL56 C325F terminase mutant had a superior replication capacity than the C325W UL56 terminase mutant in dual-infection competition assays. When the different DNA pol mutant viruses were grown in competition with the wild-type virus, in general, the mutants had a fitness advantage when the competition assays were performed under exposure to the drugs to which the mutants were resistant to. These data highlight the necessity of evaluating viral fitness in competitive assays with and without drug pressure as there is a complex interplay between viral phenotype and genotype, viral fitness, and selective drug pressure.

Importantly, the UL56 terminase mutants gained replication capacity when grown in competition with the different DNA pol mutants under LMV pressure. While the C325F was able to overgrow the DNA pol mutants, the C325WY and V236M mutants did not systematically overgrow the DNA pol mutants under LMV pressure. In our studies, we found that UL56 mutations associated with high levels of LMV resistance provide a selective advantage in the presence of LMV, and emergence of the UL56 C325F change does not lead to fitness costs for the virus, as the C325F mutant was equally fitted as the wild-type in the absence of drug pressure. In contrast to the C325F mutation, the emergence of the C325W/Y changes did result in a fitness cost for the mutant virus as the C325W and C325Y were less fitted than the wild-type virus in the absence of LMV. Thus, our data support the fact that the UL56 C325F change is the most frequent change associated with LMV resistance in vitro and in the clinic [10,23]. It can be assumed that once the UL56 C325F mutation arises in the clinic, this mutant can quite rapidly become the dominant viral variant because of its lack of fitness cost. Therefore, detection of a minor population of the UL56 C325F change by NGS should be considered as clinically relevant because of the superior competitive fitness of this UL56 mutant under LMV pressure. Close monitoring of drug resistance mutations during the treatment of an active CMV infection is needed to rapidly adjust antiviral therapy.

A recombinant virus bearing the DNA pol K513N was previously reported to have decreased susceptibility to CDV and GCV in vitro, 13- and 6.5-fold, respectively [50]. In this study, the K513N mutant showed decreased replication capacity in in vitro replication kinetic experiments. Furthermore, the K513N DNA pol mutant exhibited reduced specific enzymatic activity in comparison with the wild-type enzyme and was severely impaired in its 3′-5′ exonuclease function. Remarkably, the K513N mutant enzyme showed no decrease in susceptibility to CDV-diphosphate or GCV-triphosphate (i.e., the active metabolites of CDV and GCV, respectively) [50]. In contrast, an enzyme carrying the V715M mutation exhibited decreased sensitivity to PFA and ADV-diphosphate (active form of ADV, PMEA) but not to CDV-diphosphate nor GCV-triphosphate, which is in agreement with the phenotypic profile of the V715M mutant recombinant virus previously reported [50,51] and with our data. Therefore, drug resistance of the DNA pol V715M mutant virus is probable due to a reduced affinity of the mutant polymerase to the corresponding inhibitors. We have previously reported cross-resistance between the pyrophosphate analogues and the PME derivatives (adefovir, PMEA and PMEDAP) for different herpesviruses [52,53,54,55,56,57,58], which suggest a similar mode of interaction of herpesvirus DNA pol enzymes with PFA and the diphosphate forms of the PME derivatives.

Another important finding of our study is the lack of cross-resistance between GCV and ACV despite the fact that these two nucleoside analogues are structurally closely related (both being acyclic guanine nucleoside analogues). Ganciclovir, a derivative of ACV, differs primarily by the addition of a hydroxymethyl group at the C-2 position of the acyclic side chain. This structural modification is thought to be the reason for the greater efficacy of GCV compared to ACV against CMV. According to our data, the active GCV and ACV metabolites (GCV-triphosphate and ACV-triphosphate) appear to interact differently with the CMV DNA polymerase, with ACV-triphosphate interacting in a similar manner as PFA and the diphosphate forms of the PME derivatives, while GCV-triphosphate interacts alike CDV-diphosphate. We found that ACV and GCV select for different drug-R mutations and there is no cross resistance between ACV and GCV but well between GCV and CDV (DNA pol A987V, K513N, and F412L + Q783H) on one side, and between ACV, the PME derivatives, and PFA (DNA pol H729Y, V715M, and L773V), on the other side. Furthermore, the DNA pol K513N and DNA pol F412L + Q783H mutants clearly showed hypersensitivity to ACV and the PME derivatives.

A point of controversy is whether combination therapy should be used to prevent and/or manage emergence of drug-R CMV in the immunocompromised host. LMV resistance occurs often when the drug is used during active CMV replication, i.e., as salvage therapy [10,12,13,59]. In these patients, the major risk factor for predisposing to drug-R is the lack of CMV-specific immunity [60]. Thus, a reduction in immunosuppression should be promoted when possible [61,62]. In in vitro studies, the combination of antivirals with a different mode of action proved to be a good strategy to reduce the emergence of drug resistance in HSV-1 [63,64]. A study reporting the in vitro evaluation of classical antivirals in combination with LMV against CMV suggested that this strategy has the potential to address the resistance/cross-resistance issues [65].

Our study has some limitations. For some experiments, we lack the frequency of the viral variants at time 0 (original) as an aliquot of the inoculum was not analyzed by deep sequencing. Although we used a 50:50 ratio of the infectious virus doses in the dual-infection competition assays, we did not always have a frequency of both viruses at approximately 50% in the inoculum (original sample at day 0). This can be explained by variations in the ratios of viral DNA to infectious viral particles calculated by viral titration among the different viral stocks. In addition, not all possible combinations of the different DNA pol and terminase mutants were tested, nor did we included the R246C UL56 mutant that was reported an increased fitness in mono-culture experiments [49] while being fully sensitive to different antivirals. Another limitation is the fact that the experiments only have been done in human fibroblasts and it remains unknown how they could be extrapolated to other cell types such as epithelial or endothelial cells. Despite these limitations, our work highlights important issues that should be taken into account when studying the fitness of CMV drug-R mutants.

## 5. Conclusions

Our findings highlight the advantage of evaluating the replicative capacity of CMV drug-R mutants using dual-infection competition assays in the presence and absence of drug pressure as viral fitness is significantly affected by antivirals. In addition, our work supports the UL56 C325F terminase mutant as being the most frequently selected LMV-R virus in the clinic.

## Figures and Tables

**Table 1:** Drug susceptibility profile of CDV-R and GCV-R mutants.

Compound	Wild-Type	AD CDV-R Clone 5 (DNA Pol A987G)		AD GCV-R Clone 4C (DNA Pol K513N)	
	EC_50_ (µM)	EC_50_ (µM)	Fold-R	EC_50_ (µM)	Fold-R
CDV	0.141 ± 0.027	**1.218 ± 0.591**	**8.6**	**2.533 ± 0.744**	**17.9**
GCV	0.612 ± 0	**0.2778 ± 0.958**	**4.5**	**6.981 ± 4.986**	**11.4**
HPMPA	0.303 ± 0.233	**2.213 ± 1.196**	**7.3**	**4.0 ± 0**	**13.2**
PFA	26.9 ± 10.83	21.243 ± 0.893	0.79	26.99 ± 5.09	1.0
ADV	23.66 ± 3.0	17.89 ± 0	0.76	**4.86 ± 0.26**	**0.20**
ACV	14.93 ± 4.19	8.578 ± 0.348	0.57	**5.05 ± 0**	**0.33**
PMEDAP	9.06 ± 2.045	6.20 ± 3.88	0.68	**2.21 ± 0.44**	**0.24**
LMV	≤0.000001 ± 0	≤0.000001 ± 0	1.0	≤0.000001 ± 0	1.0

EC_50_ (50% effective concentration)—concentration required to reduce virus cytopathic effect (CPE) by 50%. Results are mean of at least 2 independent experiments. Fold resistance (Fold-R): ratio EC_50_ mutant/EC_50_ wild-type. Resistance is defined as fold-R ≥2, highlighted in red. Hypersensitivity is considered as fold-R ≤0.5, highlighted in green. Bold font is used to emphasize drug-R or drug-hypersensitivity. Abbreviations: CDV (cidofovir, HPMPC), GCV (ganciclovir), HPMPA [(S)-9-(3-Hydroxy-2-phosphonomethoxypropyl)adenine], PFA (foscarnet), ADV (adefovir, PMEA), ACV (acyclovir), PMEDAP [9-(2-phosphonylmethoxyethyl)-2,6-diaminopurine], LMV (letermovir).

**Table 2:** Drug susceptibility profile of ACV-R, PFA-R, PMEDAP-R, and HPMPA-R mutants.

Compound	Wild-Type	AD ACV-R Clone 4 (DNA Pol H729Y)		AD PFA-R Clone C (DNA Pol V715M)		AD/PMEDAP-R (DNA Pol L773V)		AD/HPMPA-R (DNA Pol F412L + Q783H)	
	EC_50_ (µM)	EC_50_ (µM)	Fold-R	EC_50_ (µM)	Fold-R	EC_50_ (µM)	Fold-R	EC_50_ (µM)	Fold-R
CDV	0.104 ± 0.022	0.128 ± 0.009	1.2	0.111 ± 0.042	1.1	0.128 ± 0.030	1.2	**0.888 ± 0.436**	**8.5**
GCV	0.535 ± 0.198	0.821 ± 0.36	1.5	0.653 ± 0.169	1.2	0.764 ± 0.063	1.4	**1.125 ± 0.563**	**2.1**
HPMPA	0.163 ± 0.155	0.123 ± 0.075	0.75	0.106 ± 0.051	0.7	0.206 ± 0.099	1.3	**1.906 ± 0.510**	**11.7**
PFA	15.73 ± 8.01	**54.67 ± 30.24**	**3.5**	**123.76 ± 18.74**	**7.9**	**96.81 ± 25.31**	**6.2**	18.60 ± 6.32	1.2
ADV	21.24 ± 8.21	**122.46 ± 26.49**	**5.8**	**77.50 ± 35.03**	**3.6**	**74.13 ± 14.84**	**3.5**	**3.15 ± 1.91**	**0.15**
ACV	15.70 ± 8.25	**>40.0 ± 0**	**>2.5**	**>40.0 ± 0**	**>2.5**	**>40 ± 0**	**>2.5**	**2.63 ± 1.78**	**0.17**
PMEDAP	10.26 ± 2.14	**73.87 ± 22.74**	**7.2**	**33.23 ± 19.91**	**3.2**	**58.95 ± 12.66**	**5.7**	**1.77 ± 0.36**	**0.17**
LMV	≤0.000001 ± 0	≤0.000001 ± 0	1.0	≤0.000001 ± 0	1.0	≤0.000001 ± 0	1.0	≤0.000001 ± 0	1.0

EC_50_ (50% effective concentration)—concentration required to reduce virus cytopathic effect (CPE) by 50%. Results are mean of 2 independent experiments. Fold resistance (Fold-R): ratio EC_50_ mutant/EC_50_ wild-type. Resistance is defined as fold-R ≥2, highlighted in red; hypersensitivity is considered as fold-R ≤0.5, highlighted in green. Bold font is used to emphasize drug-R or drug-hypersensitivity. Abbreviations: CDV (cidofovir, HPMPC), GCV (ganciclovir), HPMPA [(*S*)-9-(3-Hydroxy-2-phosphonomethoxypropyl)adenine], PFA (foscarnet), ADV (adefovir, PMEA), ACV (acyclovir), PMEDAP [9-(2-phosphonylmethoxyethyl)-2,6-diaminopurine], LMV (letermovir).

**Table 3:** Drug susceptibility profile of LMV-R mutants.

Compound	Wild-Type	UL56 C325F		UL56 C325W		UL56 C325Y Clone 5		UL56 V236M	
	EC_50_ (µM)	EC_50_ (µM)	Fold-R	EC_50_ (µM)	Fold-R	EC_50_ (µM)	Fold-R	EC_50_ (µM)	Fold-R
CDV	0.104 ± 0.022	0.116 ± 0.026	1.1	0.116 ± 0.022	1.1	0.097 ± 0.005	0.9	0.111 ± 0.024	1.1
GCV	0.535 ± 0.198	0.68 ± 0.11	1.3	0.618 ± 0.070	1.2	0.469 ± 0.118	0.9	0.577 ± 0.297	1.1
HPMPA	0.163 ± 0.155	0.27 ± 0.0.16	1.7	0.276 ± 0.164	1.7	0.223 ± 0.190	1.4	0.135 ± 0.035	0.8
PFA	15.73 ± 8.01	22.09 ± 1.20	1.4	19.65 ± 11.78	1.3	17.51 ± 12.84	1.1	22.84 ± 9.20	1.5
ADV	21.24 ± 8.21	32.28 ± 9.08	1.52	14.29 ± 3.30	0.7	17.63 ± 18.32	0.8	26.42 ± 1.65	1.3
ACV	15.70 ± 8.25	29.04 ± 9.64	1.8	23.22 ± 2.13	1.5	24.17 ± 5.63	1.5	28.74 ± 5.60	1.8
PMEDAP	10.26 ± 2.14	11.72 ± 3.25	1.1	12.14 ± 1.48	1.2	14.72 ± 9.07	1.4	13.09 ± 6.13	1.3
LMV	≤0.000001± 0	**>4**	**≥3.9 × 10^6^**	**>4**	**≥3.9 × 10^6^**	**>4**	**≥3.9 × 10^6^**	**0.021 ± 0.011**	**≥2051**

EC_50_ (50% effective concentration)—concentration required to reduce virus cytopathic effect (CPE) by 50%. Results are mean of 2 independent experiments. Fold resistance (Fold-R): ratio EC_50_ mutant/EC_50_ wild-type. Resistance is defined as fold-R ≥2, highlighted in red. Bold font is used to emphasize drug-R or drug-hypersensitivity. Abbreviations: CDV (cidofovir, HPMPC), GCV (ganciclovir), HPMPA [(*S*)-9-(3-Hydroxy-2-phosphonomethoxypropyl)adenine], PFA (foscarnet), ADV (adefovir, PMEA), ACV (acyclovir), PMEDAP [9-(2-phosphonylmethoxyethyl)-2,6-diaminopurine], LMV (letermovir).

## Data Availability

The original contributions presented in the study are included in the article/Supplementary Material. Further inquiries can be directed to the corresponding author.

## Supplementary Materials

The following supporting information can be downloaded at: https://www.mdpi.com/article/10.3390/v18070779/s1, Table S1: Purity of the CMV mutant viruses detected by NGS.

## Abbreviations

The following abbreviations are used in this manuscript:

ACV	Acyclovir
ADV	Adefovir, PMEA, 9-(2-phosphonylmethoxyethyl)-adenine
ATCC	American Type Culture Collection
CCID_50_	50% cell culture infective dose
CDV	Cidofovir, HPMPC, (S)-1-(3-hydroxy-2-phosphonylmethoxypropyl)cytosine
CMV	Cytomegalovirus
CPE	Cytopathic effect
D+	CMV-seropositive donor
DMEM	Dulbecco’s modified Eagle’s medium
DNA pol	DNA polymerase
Drug-R	Drug resistance
EC_50_	50% effective concentration, or compound concentration inhibiting 50% of viral induced CPE
FDA	Food and Drug Administration
Fold-R	Fold-resistance
GCV	Ganciclovir
HEL	Human embryonic lung
HPMPA	9-(3-hydroxy-2-phosphonomethoxypropyl)adenine
HPMPC	(S)-1-(3-hydroxy-2-phosphonylmethoxypropyl)cytosine
HSCT	Hematopoietic stem cell transplant
LMV	Letermovir
LMV-R	Letermovir resistance
MBV	Maribavir
NGS	Next-generation sequencing
PFA	Foscarnet, foscavir
PK	Protein kinase
PMEA	9-(2-phosphonylmethoxyethyl)-adenine, adefovir, ADV
PMEDAP	9-(2-phosphonylmethoxyethyl)-2,6-diaminopurine
R−	CMV-seronegative recipient
R/R	Refractory/resistant
R+	CMV-seropositive recipient
SOT	Solid organ transplant
VC	Virus control
VGCV	Valganciclovir
