# Correction: Andrei et al. Competitive Fitness of Cytomegalovirus Mutants Bearing Changes in the UL56 Terminase Subunit, Associated with Letermovir-Resistance, in Presence and Absence of Antivirals. *Viruses* 2026, *18*, 779

**DOI:** 10.3390/v18080888

**Published:** 2026-08-13

**Authors:** Graciela Andrei, Sarah Gillemot, Robert Snoeck

**Affiliations:** Rega Institute for Medical Research, Department of Microbiology, Immunology and Transplantation, KU Leuven, 3000 Leuven, Belgium; sarah.gillemot@kuleuven.be (S.G.); robert.snoeck@kuleuven.be (R.S.)

## References

In the original publication [1], there was a mistake in the References list. Two incorrect references [6] and [18] were included due to an inadvertent error during the proof stage. These two references should be replaced with the following correct citations:

6.Doss, K.M.; Heldman, M.R.; Limaye, A.P. Updates in Cytomegalovirus Prevention and Treatment in Solid Organ Transplantation. *Infect. Dis. Clin. N. Am.* **2023**, *37*, e1–e16.18.Piret, J.; Boivin, G. Clinical development of letermovir and maribavir: Overview of human cytomegalovirus drug resistance. *Antivir. Res.* **2019**, *163*, 91–105.

The authors state that the scientific conclusions are unaffected. This correction was approved by the Academic Editor. The original publication has also been updated.
