# Supplementary material for: Competitive Fitness of Cytomegalovirus Mutants Bearing Changes in the UL56 Terminase Subunit, Associated with Letermovir-Resistance, in Presence and Absence of Antivirals
Source: Viruses. 2026 Jul 15;18(7):779. doi: 10.3390/v18070779 (PMC13431593; doi:10.3390/v18070779)
Supplement: Supplementary file 1 [file viruses-18-00779-s001.zip › viruses-4317136-supplementary.pdf]

Supplementary Table S1.

Purity of the CMV mutant viruses detected by NGS

Virus	Viral gene analyzed - mutation	Variant frequency
Wild-type	UL56 / UL54	Undetectable
		Undetectable
UL56 C325F	UL56 - C325F	99.908
		99.956
UL56 C325Y*	UL56 - C325Y	84.473
		85.583
C325Y Clone 1	UL56 - C325Y	99.987
		98.529
C325Y Clone 2*	UL56 - C325Y	97.491
		95.645
C325Y Clone 3	UL56 - C325Y	99.978
		99.974
C325Y Clone 4	UL56 - C325Y	99.953
		99.968
C325Y Clone 5	UL56 - C325Y	99.963
		99.961
C325Y Clone 6	UL56 - C325Y	99.982
		99.992
C325Y Clone 7	UL56 - C325Y	99.978
		99.950
C325Y Clone 8	UL56 - C325Y	99.976
		99.946
C325Y Clone 9	UL56 - C325Y	99.957
		99.910
ACV-R Clone 4	UL54 - H729Y	99.756
		99.866
AD-169 HPMPC-R clone 5	UL54 – A987G	99.898
		99.816
AD-169 GCV-R clone 4*	UL54 - K513N	75.080
		75.474
GCV-R clone 4A	Clone 4 A1	99.979
	Clone 4 A2	99.977
GCV-R clone 4B	Clone 4 B1	99.957
	Clone 4 B2	99.989

GCV-R clone 4C	Clone 4 C1	100.000
	Clone 4 C2	99.974
GCV-R clone 4D	Clone 4D1	99.959
	Clone 4 D2	99.983
GCV-R clone 4E	Clone 4 E1	83.874
	Clone 4 E2	84.070
UL56 C325W	UL56 - C325W	99.873
		99.871
AD-169 PFA-R clone C	UL54 - V715M	99.634
		99.555
AD-169 PMEDAP-R clone 4	UL54 - L773M	99.792
		99.900
AD-169 HPMPA-R clone 6	UL54 - F412L	99.916
UL56 V236M	UL56 - V236M	99.931
		99.936

The UL56 F235F, C325Y, C325W, and V236M were kindly provided by Prof. Sunwen Chou (Research Service, Department of Veterans Affairs Medical Center , Portland, Oregon, USA).

The ACV-R Clone 4, AD-169 HPMPC-R clone 5, AD-169 GCV-R clone 4, AD-169 PFA-R clone C, AD-169 PMEDAP-R clone 4, and AD-169 HPMPA-R clone 6 were isolated in vitro in our laboratory under pressure of, respectively, acyclovir (ACV), HPMPC [cidofovir, CDV, (*S*)-1-(3-hydroxy-2-phosphonylmethoxypropyl)cytosine], ganciclovir (GCV), foscarnet (PFA), PMEDAP [9-(2-phosphonylmethoxyethyl)-2,6-diaminopurine], and HPMPA [9-(3-Hydroxy-2-phosphonomethoxypropyl)adenine]

* Since no pure populations of the viral mutants were detected by NGS, these mutants had to be purified to isolate new viral clones, which were also analyzed subsequently by NGS.
